# Differential Effects of the Home Language and Literacy Environment on Child Language and Theory of Mind and Their Relation to Socioeconomic Background

**DOI:** 10.3389/fpsyg.2020.555654

**Published:** 2020-10-29

**Authors:** Susanne Ebert, Simone Lehrl, Sabine Weinert

**Affiliations:** ^1^Department of Psychology, Norwegian University of Science and Technology, Trondheim, Norway; ^2^Department of Developmental Psychology, University of Bamberg, Bamberg, Germany

**Keywords:** language, theory of mind, home learning environment, preschool age, socioeconomic status

## Abstract

In this study, we examined differential effects of facets of the home language and literacy environment that are known to be relevant to either language development (i.e., quantity and quality of language and literacy stimulation at home) or theory of mind (ToM) development (i.e., parental mental state language), on both children’s language skills and their ToM understanding. Moreover, we investigated whether these relations are particularly relevant for children from homes with low socioeconomic status (SES) and whether they account for SES-related disparities in child language skills and ToM understanding. Using longitudinal data of a sample of 224 monolingual German preschool children (assessment of language skills at age 4;6 and 5;6 and ToM at age 5;6), we analyzed the effects of three facets of the home language and literacy environment on later child language and ToM understanding. These facets were book exposure as a measure for quantity of language and literacy stimulation at home, quality of verbal interaction, and parental mental state language assessed between ages 3 and 4. Path analyses showed that book exposure is related to both later ToM understanding and language skills at age 5;6 years; yet, this effect is mediated by earlier language skills at age 4;6 years. Furthermore, book exposure partly mediated the association between SES and language skills and, via earlier language skills at age 4;6, also the relation between SES and ToM. When focusing on children from lower SES families, book exposure and quality of verbal stimulation predicted children’s later language skills at age 4;6. Book exposure also predicted change in language skills between age 4;6 and age 5;6. Further, book exposure proved to be significantly associated with children’s ToM understanding at age 5;6 via the relation with language skills at 4;6 years. In addition, parental mental state language predicted children’s ToM understanding at age 5;6 years. Our findings provide new evidence on how different facets of the home language and literacy environment are related to ToM and language development and their interrelation as well as their SES-related disparities.

## Introduction

In preschool-age children, language is known as one of the most important predictors of children’s developing knowledge and understanding of the mental world, widely defined as theory of mind (ToM) development (Astington and Baird, 2005; Milligan et al., 2007). At the same time, environmental influences and particularly the home language and literacy environment have been shown to play an essential role in the development of both language and ToM (Hoff, 2006; Hughes and Devine, 2017). With the term “home language and literacy environment,” we refer to a wide range of facets of the home learning environment that relate to verbal communication, verbal input, and language related material (such as books) including language and literacy stimulating behavior of parents (see for similar definition, for example, Tabors et al., 2001). However, besides the close interrelation between language and ToM during children’s development, the question of how the home language and literacy environment impacts developmental progress has mainly been investigated separately for either ToM or language development. Thus, its role in the interrelation between both developmental domains has hardly been addressed. Moreover, the facets of the home language and literacy environment that are investigated in relation to language development differ from those investigated in ToM development. Against this background, the main aim of the present study was to connect these lines of research and to analyze how different facets of the home language and literacy environment that are investigated in relation to either language or ToM development are related to both children’s language and ToM as well as their interrelation.

Over and above its functional role in various domains of child development, the home language and literacy environment is also discussed as a potential mediator for differences in developmental progress in children’s language skills (e.g., Hoff, 2013) and ToM understanding (e.g., Devine and Hughes, 2016; Ebert et al., 2017) according to the family’s socioeconomic status (SES). Again, these SES-related differences in ToM and language development have rarely been connected so far. Thus, in the present study, we also investigate whether and to what extent SES-related differences in ToM and language development are explained by different facets of the home language and literacy environment. Besides, our study explores whether these facets of the home language and literacy environment are particularly relevant for children growing up in low SES families.

### Home Language and Literacy Environment and Children’s Language Development

A large body of research suggests that providing children with the experience of a varied and rich language and literacy exposure at home, such as sharing books, involving children in discourse, and using child-directed speech including recasts, extensions, and reformulations of the child’s utterances, promotes children’s language and literacy development (e.g., Hoff-Ginsberg and Shatz, 1982; Sénéchal et al., 1998; Burgess et al., 2002; Mol and Neuman, 2014). Thereby, quantitative and qualitative aspects of language and literacy exposure are to be distinguished. The quantity of language and literacy exposure refers, amongst others, to children’s more informal experiences with literacy and literacy-related material (e.g., availability of books at home, frequency of shared book reading) and has been shown to be related to children’s language and literacy development (e.g., Sénéchal and LeFevre, 2002; Mol and Bus, 2011; Lehrl et al., 2012). The quality of language exposure during shared book reading is also highly relevant to children’s language development; in particular, a varied and complex language input, a high level of decontextualization from the here and now, asking open-ended questions, elaborating on the child’s comments, and interacting in a responsive way that adapts to the needs of the child are related to child’s language development (e.g., Reese and Cox, 1999; van Kleeck, 2003; Rowe, 2012; Lehrl et al., 2013; Mol and Neuman, 2014). For instance, Lehrl et al. (2012) showed that both book exposure and the quality of parent-child interactions during a shared book reading situation (e.g., asking open-ended questions, using stimulating language) measured when children were 3 years of age explained variance in children’s language development during the next year; yet, the correlations between relevant facets of the home language and literacy environment were only low to medium, and differential effects were observed for vocabulary and grammar.

### Home Language and Literacy Environment and Children’s ToM Development

As for language development, rich experiences in language input at home are related to children’s ToM understanding and development. For instance, studies with deaf children of hearing parents showed that these children are delayed in ToM development; in contrast, deaf children of deaf parents are not (e.g., Peterson and Siegal, 2000). This result is explained by differences in the children’s home language and literacy environment: Hearing parents are not proficient in sign language and thus cannot provide a comparatively rich and stimulating home language and literacy environment. Moreover, longitudinal and training studies including typically developing children support the assumption that verbal interaction and language input promote children’s ToM development (Ruffman et al., 2002; Lohmann and Tomasello, 2003). In particular, a specific type of verbal interaction, namely mental state language, has been suggested to support children’s developing ToM (Ruffman et al., 2002; Gola, 2012; Ebert et al., 2017). Mental state language refers to language that is used to talk about mental states and processes (Bretherton and Beeghly, 1982; Antonietti et al., 2006; Olson et al., 2006). It includes verbal expressions that refer to mental states such as desires, intentions, or knowledge (e.g., “want,” “belief,” “knowledge,” “memory”) as well as talk about mental entities in general, even without explicitly naming mental states.

Verbal interactions between parents and their children vary with respect to the frequency and the way in which they talk about mental states (e.g., Ruffman et al., 2002; Peterson and Slaughter, 2003). Thus, parents’ mental state language can be conceptualized as a specific aspect of the quality of the home language and literacy environment, and differences in this facet have been shown to relate to children’s ToM development. For example, Ebert et al. (2017) demonstrated that the children of parents with a higher preference for using mental state language in everyday situations show faster growth in ToM understanding from ages 3 to 5 compared to their peers whose parents preferred mental state language less.

A recent meta-analysis (Tompkins et al., 2018) showed that particularly mental state talk about cognitive mental states as well as mental state talk that explains and elaborates on mental states was most predictive for children’s ToM understanding. Moreover, the relations were more pronounced in studies observing mental state talk in a book reading context or when it was self-reported and less when reminiscing or play situations were observed. Further, the correlation between parental mental state talk was higher for children’s false-belief understanding than for their emotion understanding. False-belief understanding comprises children’s understanding that mental states may differ from reality (and thus can be false) but nevertheless motivate peoples’ behavior. Typically, such an understanding develops between 3 and 6 years of age. False-belief understanding is widely accepted as one of the most critical steps in children’s ToM development (Wellman et al., 2001).

### Specific Effects of the Home Language and Literacy Environment?

In general, parental mental state language shares features with high-quality verbal interactions. For instance, mental state language is usually decontextualized language as mental states are not visible; when talking about what people think or know, this goes beyond the here and now. Moreover, mental state talk is often embedded in complex grammatical sentence structures, known as sentential complements (De Villiers and Pyers, 2002). Furthermore, mental state talk that elaborates and explains mental states comprises features of high-quality verbal interactions as this kind of talk often implies complex grammatical structures and is related to the quantity and quality of language stimulating verbal interactions in general (see also Hoff and Naigles, 2002; Ebert, 2011). Thus, parental mental state language might be conceptualized as high-quality verbal interaction; at the same time, high-quality verbal parent-child interactions may include talking about mental entities. Thus, for example, involving children in discussions about picture books, asking open-ended questions, and using decontextualized talk often means asking children about their own or the story protagonists’ mental states. Moreover, the content of stories or books frequently refers to mental states such as the goals, intentions, or feelings of the story characters; thus, providing books and shared picture book reading may also support children’s understanding of mental states (Astington and Pelletier, 1996; Dyer et al., 2000; Farkas et al., 2020).

However, until now research has rarely connected the more general facets of home language and literacy environment with the more specific mental aspects of the home language and literacy environment. Moreover, besides the close interrelation between language and ToM in development, there is not much research that investigates the interrelation between these specific facets of the home language and literacy environment and their effects on both ToM *and* language development.

One of the rare studies that connects the home language and literacy environment with children’s ToM and language was conducted by Boerma et al. (2017). This study included children at the age of 9–10 years and showed that a measure of book exposure at home was likewise related to both children’s advanced language competencies and their ToM understanding. However, the children were already in primary school, and the relations between measures were only assessed concurrently. Moreover, measures of the ToM-specific home language and literacy environment, i.e., parents’ mental state language, were not included in the study.

A study by Adrian et al. (2005) with 4–5-years-old children, in contrary, included parents’ mental state language. The authors analyzed how mothers’ mental state language during a book reading session and the frequency of book reading were related to children’s ToM development. Interestingly no correlations were found between the frequency of joint book reading at home and the number of words or mental state terms (variety and quantity) the mother used during picture book reading. However, both the frequency of joint picture book reading and the usage of mental state terms were related to children’s false-belief understanding, even after controlling for parents’ education and age of the children. Mothers’ usage of mental state terms during picture book reading even explained additional variance in false-belief understanding after accounting for the frequency of joint picture book reading and the number of other words used during picture book reading. However, although the authors included more general facets of the home language and literacy environment *and* specific mental facets, they focused only on ToM understanding but not on children’s language skills and how these are related to the various facets of the home language and literacy environment. Thus, it remains unclear how the various facets of the home language and literacy environment are related to language in comparison to ToM development and how they might impact the relation between children’s language and ToM. Moreover, as their study was cross-sectional, it cannot provide information on how the various facets of the home language and literacy environment affect the relation between language and ToM in development.

Against this background, one aim of the present study was to investigate whether there are specific relations between child language and the quantity as well as quality of language stimulating verbal interactions on the one hand and between ToM and parental mental state talk on the other hand or whether both indicators of the home language and literacy environment are comparably related to both domains of development and may even account for their interrelation.

### Home Language and Literacy Environment as a Mediator and Moderator of SES-Related Disparities in Language and ToM Development

Children from low SES families, i.e., from families with low income and/or low education, often perform below their peers from higher SES families on cognitive measures and academic achievement (e.g., Bradley and Corwyn, 2002). Accordingly, significant SES-related disparities have been documented for language (see Hoff, 2013) and ToM development (see Devine and Hughes, 2016).

One mechanism or pathway explaining the association between SES and children’s language development is via SES-related differences in language input and the quality of verbal interactions (Huttenlocher et al., 2010; Fernald and Weisleder, 2011; Hoff, 2013; Mol and Neuman, 2014; Pace et al., 2017). Following this assumption, parents with a higher SES provide their children with a comparatively richer home language and literacy environment than parents with a lower SES. They not only offer more books as well as literacy related activities to their children (z.B. Bradley et al., 2001; Fletcher and Reese, 2005; Leseman et al., 2007; Crosnoe et al., 2010), but they also speak more often with their children and use more complex and varied language (Hart and Risley, 1992; Arriaga et al., 1998; Huttenlocher et al., 2002, 2010).

Studies show that differences in the home language and literacy environment can at least partly explain SES-related differences in language skills (Huttenlocher et al., 2002; Hoff, 2003; Huttenlocher et al., 2010; Ebert et al., 2013; Mol and Neuman, 2014). However, the relations between SES, home language and literacy environment, and language development are more complex, and not all studies find this mediating effect. Thus, for example, whereas Huttenlocher et al. (2002) showed that differences in language input including complex grammatical structures accounted for SES-related differences in children’s grammar, Weinert and Ebert (2013) did not find a mediating effect of a general indicator of the home language and literacy environment. The partially controversial results suggest that SES-related differences in child language might be differentially related to specific facets of the home language and literacy environment that may account for SES-related differences in child language (for a similar suggestion see also Rowe, 2012).

For ToM development, it is even less clear whether parental (mental state) language accounts for individual differences associated with SES. Only a few studies focused on SES, parental mental state language, and ToM. However, in their meta-analysis, Devine and Hughes (2016) found that the relation between SES and ToM is not completely explained by differences in parental mental state language. Moreover, in a longitudinal study including more than 120 preschoolers, Ebert et al. (2017) did not find differences in parental mental state language according to SES. Thus, parental mental state language did not explain individual differences in the children’s ToM development between 3 and 5 years that were associated with SES background. However, the study results showed that, depending on SES, different types of parental mental state language were associated with ToM development: Whereas for children from higher SES backgrounds parents’ preference for elaborated mental state language that explains and elaborates on these mental states was associated with children’s ToM understanding, for children from lower SES families it was in particular the parents’ preference for more basic mental state language, i.e., mental state language without broad elaborations and explanations of the mental states, that promoted children’s ToM development. This result suggests that parental (mental state) language may not affect ToM development in the same way for all children.

It is also very likely that the effects of the home language and literacy environment on children’s language development are moderated by social background. Thus, correlations between facets of the home language and literacy environment and language development are often documented particularly for low SES samples (e.g., Storch and Whitehurst, 2001; Pan et al., 2005; Mistry et al., 2008; Vernon-Feagans and Bratsch-Hines, 2013; Hirsh-Pasek et al., 2015; Rowe et al., 2017) and seem more pronounced in children from lower SES families (e.g., Bradley et al., 2001; Baydar et al., 2014; Shahaeian et al., 2018). Moreover, a study by Weinert et al. (2012) found differences in the home language and literacy environment to be particularly relevant to vocabulary development in a group of children with less advanced language skills at age 3 as compared to the children with more advanced language skills.

Against this background, another aim of our study was to investigate how different facets of the home language and literacy environment are related to SES and whether they account for differences in language and ToM development that are associated with SES. In addition, we analyze whether the effects of the home language and literacy environment are particularly pronounced in children from lower-SES families.

### The Present Study

Previous studies have shown that various facets of the home language and literacy environment are connected to either language or ToM development. Moreover, ToM understanding and language skills are related in development (e.g., Milligan et al., 2007; Ebert, 2015, 2020). Against this background we investigated the relation of different facets of the home language and literacy environment to both language skills *and* ToM understanding as well as to their relation in development.

Drawing on longitudinal data, we analyzed how three facets of the home language and literacy environment of 3–4-year-old children are related to their language skills *and* ToM understanding two years later, i.e., at the age of 5–6 years, and how language skills at age 4–5 years mediate these relations. The three facets of the home language and literacy environment we included in our study are (a) book exposure as a proxy for the quantity of language and literacy stimulation at home, (b) quality of verbal interaction during shared picture book reading as a measure of the quality of verbal stimulation at home, and (c) parental mental state language as a measure of a specific mental facet of the home language and literacy environment that has been shown to be associated with children’s ToM development. In particular, we addressed the following research questions:

1. Is the general quantity and quality of the home language and literacy environment in the development related to language skills and also to ToM understanding?
2. Is parental mental state talk specifically related to ToM understanding even after accounting for more general facets of the home language and literacy environment, or is parental mental state talk a subdimension of overall language stimulation and thus also related to children’s language development?
3. Are the various facets of the home language and literacy environment directly related to ToM or only indirectly via children’s language development?

Concerning SES, we expected the various facets of the home language and literacy environment to explain SES-related differences in language skills and ToM understanding. Further, we assumed that the effects of the various facets of the home language and literacy environment are particularly significant for children from lower SES families. In particular, we addressed the following research questions:

1. Are the various facets of the home language and literacy environment associated with SES, and do they mediate SES-related disparities in language skills and ToM understanding?
2. Are the effects of the home language and literacy environment particularly pronounced in low-SES families?

## Materials and Methods

### Sample and Procedure

The present sample was part of a more comprehensive German longitudinal study on child development and educational processes. The study was funded by the German Research Foundation, and compliance with ethical standards was approved by the review process. Appropriate consent to take part in this study was obtained from parents, and all information provided was voluntary. Data collection started in 2005, including 547 children from 97 preschools in rural and urban areas of Bavaria and Hesse, Germany. All children of one randomly selected group within each preschool who would enter school in 2008 were asked to take part in the study. Thus, at the first measurement point, children were about 3 years old. Various measures of home and preschool environment, as well as of children’s development, were collected every half year.

**FIGURE 1:**
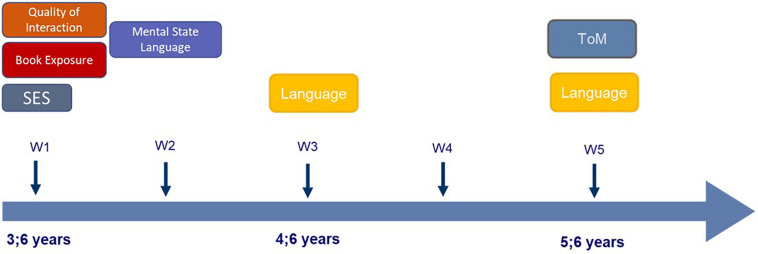
Overview of the included measures at the various waves (W).

The present study draws on measures collected at assessment waves 1, 2, 3, and 5. At Wave 1, measures of SES and the quantity (book exposure) and quality (quality of verbal interaction) of the home language and literacy environment were assessed. At Wave 2, parents were presented with the instrument for measuring parental mental state language. The outcome measures for child language and ToM were assessed at Wave 5, and we included also child language at Wave 3 as a potential mediator that might explain the relation between home language and literacy environment and ToM development (see Figure 1 for an overview).

### Participants

The present study included the subsample of 267 children, who were – by design – assigned to the subgroup of children who received ToM measures at Wave 5. These children attended preschools in Bavaria. We included only monolingual children (i.e., children whose parents were native German speakers) as we focus on relations including language development and language stimulation. Thus, in total, 224 children (51.3% boys) took part in the present study. At Wave 1 of the study, these children were, on average, 41.87 months (3;6 years; *SD* = 3.98 months) old.

### Measures

#### Family Variables

Families were visited every year at their home and were presented with a computer-assisted personal interview. In this interview, parents were asked for information on various SES indicators such as education and occupation as well as on educational practices and child characteristics. In the middle of the interview, parents were given a picture book and asked to share it with their child. This interaction was observed by a trained interviewer, who rated the quality of verbal interaction (see below). After the parent and child signaled that they had finished the joint picture book reading, the interview was continued. At the end of the visit, parent and child received a small gift.

Parents were also given a questionnaire every half a year asking for further child and family-related variables as well as educational practices, which they should send back by mail.

##### Socioeconomic status (SES)

As a general measure for family SES we referred to the International Socio-economic Index of Occupational Status (ISEI; Ganzeboom et al., 1992). The ISEI is based on international data about education, income, and prestige of various occupations. Possible levels range from 16 (e.g., cleaner, unskilled farm worker) to 90 (e.g., judge in a court of law). To avoid underestimating the family’s SES, we used the highest ISEI (HISEI) of the parents.

##### Quantity of language stimulation within the family – book exposure

To measure the quantity of more informal language and literacy-related interactions within the family, an index for book exposure was created as a proxy. Therefore, the answers parents gave in the questionnaire at Wave 1 on how frequently they read to their child (1 = never to 5 = daily), on the number of books in the household (1 = up to 30, 2 = up to 100, 3 = up to 200, and 4 = more than 200 books), and on the number of children’s books in the household (1 = up to 10, 2 = up to 20, 3 = up to 30, and 4 = more than 30 books) were used. The items were first standardized to represent a range of 0–1 and then averaged. Cronbach’s alpha was 0.68.

##### Quality of verbal parent-child interaction

**FIGURE 2:**
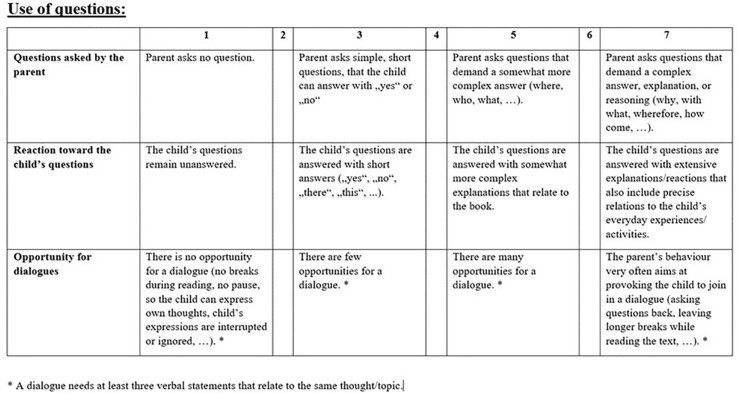
Example of the qualitative characterization of the three indicators (“questions asked by the parent,” “reaction toward the child’s questions”, “opportunity for dialogues”) of the subscale “use of questions.”

To gather information on the quality of verbal parent-child interaction during joint picture book reading the Family Rating Scale (FES; Kuger et al., 2005) was used. Therefore, a semi-standardized picture book reading situation between the primary caregiver (96% mothers) and the child was conducted at the family’s home (see above). The picture book used at Wave 1 was about a family’s visit to a circus and designed within the project. Thus, it was unknown to the parent and the child. The parents were advised to share this book with their child as they usually do in joint picture book situations. The quality of this interaction was rated by a trained observer on 11 subscales. Each subscale includes up to three indicators that are rated on a 7-point scale (1 = low quality to 7 = high quality). The scale-levels 1 (low quality), 3 (minimal quality), 5 (high quality), and 7 (excellent quality) are qualitatively characterized and described to facilitate and standardize the ratings. A subscale score was calculated as the mean across the indicators. For instance, the subscale “use of questions” comprises three indicators: “questions asked by the parent,” “reaction toward the child’s questions,” “opportunity for dialogues” (see Figure 2 for an example of the qualitative characterizations). For the present study, the mean across those 6 subscales referring to language and literacy was used to represent an indicator for verbal parent-child interaction quality (see Table 1 for a brief description). Cronbach’s alpha was 0.65. Beforehand raters had been trained to a criterion of 90% agreement (± 1) to a gold standard of a master rating. Ten percent of observations were double coded by two independent raters; rater agreement was good (ICC = 0.78). The scale was linear transformed to a range of 0–1.

**TABLE 1:** Description of the 6 subscales of the family rating scale referring to language and literacy used as an indicator for verbal parent-child interaction quality.

Subscale	Description (high quality continuum)
Level of distancing	Caregiver initiates dialogue, refers to the pictures in the book and includes distant (not visible) aspects of the situation.
Non-verbal behavior	Caregiver shows positive gesture and body language. He or she pays attention and shows interest toward the child’s utterances and behavior.
Use of questions	Caregiver uses complex questions (e.g., “why?”) and responds to child’s questions elaborately.
Level of speech	Caregiver uses rare words, provides correct, complex, and appropriate language in terms of vocabulary and syntax to the child.
Phonological cues	Caregiver correctly articulates phonemes, emphasizes syllable segmentation, and encourages the child to do rhymes by his/her own.
Participation in dialogue	Child gets the opportunity to participate in or even guide the dialogue between caregiver and child.

##### Parental mental state language

To assess parents’ mental state language, parents filled in a questionnaire that included four vignettes (In the Kitchen, Dad’s Birthday, Lost Keys, The Empty Flask) of the Maternal Mental State Input Inventory (MMSII; Peterson and Slaughter, 2003) at Wave 2. These four vignettes were chosen because they had a particularly pronounced cognitive emphasis. Each vignette depicts an episode of everyday interactions (e.g., baking a cake together; searching for lost keys) between a mother and a 4-year-old child and is followed by four possible options what a parent could say in the described situation: Two of these options are mental. One is an Elaborated Mental State (EMS) option, and the other is a Non-Elaborated Mental State (NEMS) option. In the EMS option, the mother explicitly names a mental state (e.g., surprise) and explains or elaborates this mental state while giving further information (e.g., “Dad doesn’t know what is inside the box, because he can’t see inside the box now that it is all wrapped up. If you tell him, he won’t be surprised when he opens it”). In the NEMS option the mother also explicitly mentions the mental state but does not further elaborate on it or explain it (e.g., “John, don’t tell Daddy what we’ve got him. We want him to be surprised on his birthday.”). The two other options were mainly included as distractors to enable the respondent to choose between other conversational strategies that were non-mental but comparable in lengths. Thus, one non-mental option was an elaborated one and the other a non-elaborated one (see Peterson and Slaughter, 2003, for more information). For each vignette parents were asked to rank order those four options according to the answer they would themselves most likely give to their child in such a situation (i.e., 1 to their most likely answer, 2 to their second likely answer, and so on). For statistical analyses these hierarchical rankings were converted into preference scores ranging from 4 (highest preference) to 1 (lowest preference) and mean scores for NEMS and EMS options over the four vignettes were calculated. For instance, if parents choose the EMS option in all four vignettes as their first preference and the NEMS option always as second preference, they receive a mean score of 4 for EMS and a mean score of 3 for NEMS. The reliability of the MMSII, even when using only those four vignettes, is moderate (for more information see Ebert et al., 2017).

#### Child Variables

Child language and ToM were measured together with other cognitive tests at one of three testing sessions per wave in a separate room of the child’s preschool. The individual testing session lasted about 30 minutes and was conducted by a trained research assistant. Parents had provided informed consent beforehand, and the child had the opportunity to withdraw from testing at every time point during testing. After each testing session children received a small gift from the research assistant.

##### Language skills

At Wave 3 and Wave 5 children were tested for their receptive vocabulary and grammar.

For assessing receptive vocabulary a German research version of the Peabody Picture Vocabulary Test (PPVT; Dunn and Dunn, 1981) was administered. In this test, per item the child has to select the appropriate picture out of four pictures that corresponds to a verbally presented lexical item. Items were presented in order of increasing difficulty and according to the original PPVT procedure, testing was stopped when the child’s response to 6 or more items within a set of 12 items (last set 7 items) was incorrect. Each correct response was scored as 1 point (max. 175).

For measuring receptive grammar, a shortened German research version including 48 items of the Test for the Reception of Grammar (TROG, Bishop, 1983/1989; German Version: TROG-D, Fox, 2006) was implemented. In this test children are asked to select (out of 4 choices) the respective picture corresponding to a stimulus sentence with grammatical structures of increasing grammatical complexity. Our version tested for all grammatical structures included in the original test, but with 2 (except for the first three sets) rather than 4 items per structure. Each correct answer scored 1 point, and a maximum of 48 points could be received.

At Wave 3 the correlation between vocabulary and grammar was *r*(204) = 0.53 and at Wave 5 it was *r*(187) = 0.44. These correlations did not differ significantly between waves. We z-standardized and averaged the scores of vocabulary and grammar at each wave as a general language indicator. The stability of this indicator across waves was numerically higher (*r* = 0.69) than the stability of its components, i.e. of vocabulary (*r* = 0.56) and grammar (*r* = 0.57).

##### Theory of mind

At Wave 5 children received one first-order unexpected content false-belief task (based on Perner et al., 1987) and one second-order false-belief task (Sullivan et al., 1994). Both tasks were presented as narrated stories and were acted out with small figures and props.

For the unexpected content false-belief task the child was shown a familiar, pictorially-labeled container (e.g., an egg box) and was asked what she or he thinks it might hold. Afterwards the child was shown that there was something unexpected (e.g., a toy animal) in the container. Then a naive protagonist (P) was introduced and the false-belief test question was asked: “What does P think is in the box?” A control questions (“Did P look inside the box?”) had also to be answered correctly to pass. The child was also given a test question about own belief (“Before you had a look inside the box, what did you think was inside?”). Total first-order false-belief scores range from 0 to 2 (*M* = 1.31, *SD* = 0.70).

In the second-order false-belief task children were told a story about a boy (Peter) who had seen his actual birthday present (a dog) unbeknownst to his mother, who has told him that he will receive a different present (a toy). The mother had a phone call with Peter’s grandma talking about Peter’s present. While the child listened to the story, she or he is asked two control questions (“What has Mum really got Peter for his birthday?”; “What did Peter’s Mum say to him that he got for his birthday?”) and three test questions: a knowledge-access first-order question (“Does Mum know that Peter saw the dog?”), a knowledge-access second-order question (“When Grandma rings up and asks if Peter knows what his present is, what will Mum say?”), and a false-belief second-order question (“What present will Peter’s Mum tell Grandma that Peter thinks he is getting?”). For each test question children could earn 1 point, thus in total 3 points for the second order task (*M* = 1.76, *SD* = 1.10). Again, control questions had to be passed along with the test questions or the item was failed.

Scores on the first-order and second-order task were summed to form a comprehensive ToM score [*r*(186) = 0.34]. Thus, the total ToM score could range from 0 to 5 points.

### Analytic Plan

In order to address our research questions we ran path analyses using Mplus 7 (Muthén and Muthén, 2012). To explore the relations between the various facets of the home language and literacy environment and children’s language skills and ToM understanding, we carried out two path analyses. First, we conducted a path analysis, where we regressed language and ToM at age 5;6 years (Wave 5) on all facets of the home language and literacy environment simultaneously to analyze whether there are specific effects of the different facets. Further, to investigate how the home language and literacy environment affects the relation between language and ToM in development and whether only indirect effects of the various facets of the home language and literacy environment on ToM exist via earlier language skills, we added language at age 3;6 years as a mediator into the analysis.

To explore further whether facets of the home language and literacy environment mediate the relation between SES and the children’s language skills and/or their ToM understanding, we conducted a mediational analysis including the family’s SES into the model.

Finally, to analyze whether the home language and literacy environment is particularly important for children growing up in families with a lower SES, we ran the path model including all facets of the home language and literacy environment only for children from relatively lower SES families (i.e., those scoring below the median of the HISEI of the whole sample). As our focus is on children from lower SES families, we only report results of this group. However, for integrity the results of a multiple group analysis including also the children from higher SES families can be found in the Supplementary Material (Figure A).

Due to the longitudinal study design, we had dropouts over time as well as missings due to illness or refusal to take part in the study at certain measurement points. However, dropout at Wave 5 was still small, and the children who had no valid score in ToM at Wave 5 did not differ significantly (*p* > 0.05) in any of the variables included in the study from those children with valid data.

Furthermore, not all parents sent back the questionnaire that included the vignettes of the MMSII, and some answers were invalid (e.g., parent gave only one rank) and had to be excluded from the analyses. The children of parents with no valid data on the MMSII differed significantly from the other children in HISEI [*t*(119.99) = 2.19, *p* < 0.05], receptive grammar at age 4;6 years [*t*(79.88) = 2.13, *p* < 0.05], and marginally significantly in book exposure [*t*(39.96) = 1.80, *p* < 0.10]. There were no other significant differences in any of the variables included.

To account for missing data, we used a full information maximum likelihood (FIML) approach, which is recommended especially in case of incomplete outcome variables and results in less biased parameter estimates than other methods (Graham, 2003; Enders, 2013). FIML is superior to listwise deletion, pairwise deletion, and single response imputation, especially in small sample sizes and when missing is at random (Enders and Bandalos, 2001). Due to the small sample size we also calculated bias-corrected bootstrapping confidence intervals for all models. The results of this procedure were very similar to the standard models reported below.

## Results

Table 2 shows descriptive information for all variables included in the study, and Table 3 presents the correlations among our key measures.

**TABLE 2:** Descriptives for the key measures of the study.

	*N*	*M*	*SD*	Min	Max
**Child variables**					
Age at Wave 1 (in months)	216	41.87	3.98	34	49
Age at Wave 3 (in months)	205	53.62	3.95	46	61
Age at Wave 5 (in months)	187	65.57	3.98	58	74
PPVT, Wave 3 (age 4;6 years)	202	54.53	18.29	0	109
TROG, Wave 3 (age 4;6 years)	204	30.82	6.35	10	45
PPVT, Wave 5 (age 5;6 years)	178	78.34	19.39	13	139
TROG, Wave 5 (age 5;6 years)	187	36.84	4.38	23	45
ToM, Wave 5 (age 5;6 years)	186	3.07	1.49	0	5
**Family variables**					
HISEI	223	52.64	15.82	20	88
Book exposure	186	0.74	0.21	0.11	1.00
Quality of verbal interaction	216	0.63	0.09	0.25	0.85
Mental state language (EMS)	157	2.94	0.58	1.75	4.00
Mental state language (NEMS)	157	2.96	0.60	1.25	4.00

**PPVT, Peabody Picture Vocabulary Test; TROG, Test for the Reception of Grammar; ToM, Theory of Mind; HISEI, Highest International Socio-Economic Index of occupational status in the family; EMS, elaborated mental state language; NEMS, non-elaborated mental state language.**

**TABLE 3:** Correlations between key variables.

	1.	2.	3.	4.	5.	6.	7.	8.
1. HISEI	–	0.39**	0.33**	–0.11	0.10	0.28**	0.35**	0.21**
2. Book exposure			0.21**	0.01	0.16*	0.33*	0.32**	0.23**
3. Quality of interaction				–0.11	0.17*	0.10	0.10	0.18*
4. Mental language (EMS)					−0.47**	–0.03	–0.11	–0.07
5. Mental language (NEMS)						0.11	0.14	0.18*
6. Child language (4;6 years)							0.69**	0.46**
7. Child language (5;6 years)								0.45**
8. ToM (5;6 years)								

ToM, Theory of Mind; HISEI, Highest International Socio-Economic Index of occupational status in the family; EMS, elaborated mental state language; NEMS, non-elaborated mental state language. **p < 0.01, *p < 0.05, ^+^p < 0.10.

First, concerning the intercorrelations between SES and the facets of the home language and literacy environment, Table 3 shows that the HISEI was moderately related to the quantity (book exposure) and quality (verbal interaction during joint book reading) indicators of the home language and literacy environment. In contrast, SES was not related to either of the two indicators of parental mental state language. Moreover, the various facets of the home language and literacy environment were only slightly interrelated. Thus, the correlations between book exposure, quality of verbal interaction, and non-elaborated parental mental state language were all low (*r* = 0.16–0.21, *p* < 0.05), and neither book exposure nor quality of verbal interaction was related to elaborated mental state talk. The high negative correlation between NEMS and EMS was due to the fact that parents had to rank order the options; thus, if they choose, for example, NEMS as the first rank, EMS is given a lower number. Therefore, NEMS and EMS are not independent of each other.

Second, Table 3 also shows that, as expected, language skills at Wave 3 and Wave 5 were correlated with ToM understanding, and both language and ToM were related to SES. However, numerically the correlation between SES and language was higher than the correlation between SES and ToM.

### Relation Between the Different Facets of the Home Language and Literacy Environment and Child Variables

Table 3 shows that the correlations of the various facets of the home language and literacy environment with children’s language skills in comparison to their ToM understanding differ in magnitude. Although book exposure was related to language and ToM, ToM was numerically less related to book exposure (*r* = 0.32 vs. *r* = 0.23). Concerning the quality of verbal interaction, however, it was ToM understanding that showed a small but significant correlation with the quality of verbal interaction (*r* = 0.18, *p* < 0.05), but not language skills. None of the two measures of parental mental state talk was related to language, but non-elaborated mental state language was associated with ToM understanding to a small but significant degree (*r* = 0.18, *p* < 0.05).

**FIGURE 3:**
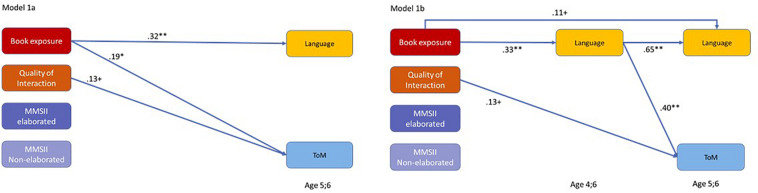
Path models showing the relations between facets of the home language and literacy environment and children’s later language as well as theory of mind (ToM). Depicted are only paths that were significant on *p* < 0.10. MMSII = Maternal Mental State Input Inventory. **p < 0.01, *p < 0.05, ^+^*p* < 0.10.

In the first path model, we tested how the different facets of home language and literacy environment are related to later language skills and ToM understanding, when considered simultaneously (see Figure 3). In Model 1a, we included only language and ToM at Wave 5, whereas in Model 1b, we also entered language at Wave 3 to investigate whether early language mediates the relations between home language and literacy environment and ToM.

As Figure 3 shows, when all facets of the home language and literacy environment are considered simultaneously, only book exposure predicted later language skills, whereas book exposure and quality of verbal interaction were related to later ToM understanding. None of the indicators of parental mental state language was correlated with ToM or language when considering the more general indicators of quantity and quality of the home language and literacy environment simultanously (see Model 1a). Model 1b demonstrates that the effect of early book exposure on later ToM was completely mediated by early child language and thus had only an indirect effect on ToM (β = 0.13, *p* < 0.01). In contrast, when considering early child language as a possible mediator, the significant direct effect of the quality of verbal interaction on later ToM as well as the direct effect of book exposure on later language remained. Note that the quality of verbal interaction neither affected language at age 4;6 nor at age 5;6 directly or indirectly in the model.

### Relations Between SES, Child Language, and ToM: Mediating Role of the Home Language and Literacy Environment

In the next step, we investigated whether facets of the home language and literacy environment can explain the relation between SES and children’s later language skills and ToM understanding by including the family’s HISEI as an indicator for SES into the model. We specified the direct effects of HISEI on ToM and language measures as well as indirect effects via the various facets of the home language and literacy environment. According to Baron and Kenny (1986), we included only book exposure and quality of interaction in the model as SES was not related to parental mental state language in our study (see Table 3).

**FIGURE 4:**
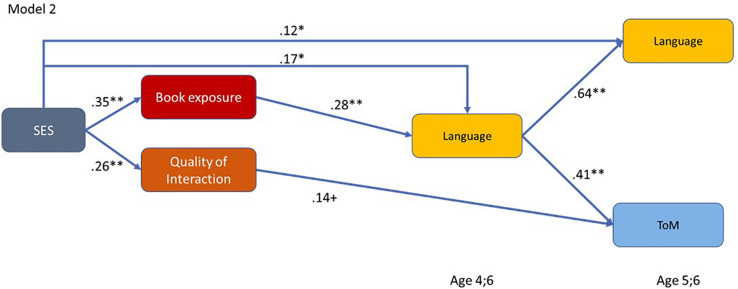
Path model showing the relations between SES, facets of the home language and literacy environment, and children’s later language skills and ToM understanding. Depicted are only paths that were significant on *p* < 0.10. **p < 0.01, *p < 0.05, ^+^*p* < 0.10.

Figure 4 shows direct paths of SES on later language, even when considering book exposure and quality of verbal interaction in the model. However, the relation was reduced (compared to the relation shown in Table 3). Moreover, we found an additional indirect effect of SES on language skills at age 4;6 and 5;6 via book exposure though not via our measure of the quality of verbal interaction. The indirect effect of SES on language at age 5;6 via book exposure and language age 4;6 was β = 0.04 (*p* < 0.05). This result indicates that the relation between SES and language was partly mediated by book exposure.

Figure 4 also shows that, in contrast to child language, SES was not directly related to ToM after considering book exposure, quality of verbal interaction, and child language at age 4;6. The relation between SES and later ToM (*r* = 0.21, *p* < 0.01) was completely mediated, especially via language skills at age 4;6 (β = 0.07, *p* < 0.05) and via book exposure and language skills at age 4;6 years (β = 0.02, *p* < 0.05).

### Effects of the Home Language and Literacy Environment in Children From Low SES Backgrounds

**FIGURE 5:**
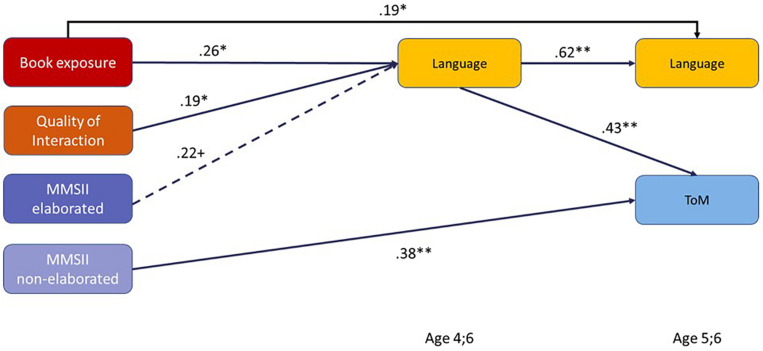
Path model showing the relations between facets of the home language and literacy environment and children’s later language skills and ToM understanding for children from low-SES families. Depicted are only paths that were significant on *p* < 0.10. MMSII, Maternal Mental State Input Inventory. **p < 0.01, *p < 0.05, ^+^*p* < 0.10.

To analyze whether the demonstrated relations also hold for the group of children from comparatively lower SES families and may even be particularly pronounced, we ran a model similar to Model 1b (see Figure 3) for children from lower SES families only (see Figure 5).

As Figure 5 shows, there was an overall comparatively high impact of the home language and literacy environment in the lower SES families. We found the quantity and the quality of the home language and literacy environment to be related to the children’s later language skills in this group. Also, one specific mental facet of the home language and literacy environment, the non-elaborated parental mental state language, showed a direct path to later ToM, even when the quantity and quality aspects of the home language and literacy environment and earlier language skills were considered simultanously.

Moreover, we found indirect effects of the quality and quantity of the home language and literacy environment on ToM and language skills at age 5;6 via the children’s earlier language skills at age 4;6. With regard to ToM understanding at age 5;6 book exposure had an indirect effect of β = 0.11 (*p* < 0.05) and the quality of verbal interaction had an indirect effect of β = 0.08 (*p* < 0.10) via language skills at age 4;6. With regard to language skills at age 5;6, book exposure had an indirect effect of β = 0.16 (*p* < 0.05) and the quality of verbal interaction had an indirect effect of β = 0.12 (*p* < 0.10) via language skills at age 4;6.

The impact of the home language and literacy environment on children’s language skills and ToM understanding in the higher SES families was much less pronounced (see Supplementary Figure A for the results of a multiple-group analysis that differentiates between lower and higher SES families). In the higher SES families only book exposure showed a significant positive effect on language skills at age 4;6.

## Discussion

In the following, we discuss our results and their implications along with the different research questions of our study. We will mainly focus (a) on the direct and indirect effects of the different facets of the home language and literacy environment on children’s language and ToM development, (b) on SES-related differences in language and ToM development and the mediating role of the home language and literacy environment, (c) on the specific pattern of results concerning parental mental state language, and (d) on the effects of the home language and literacy environment of children from less advantaged homes.

### Specific Effects of the Various Facets of the Home Language and Literacy Environment on Language and ToM

A unique aspect of our study was that we connected research on the relation between the home language and literacy environment and children’s language development with research on the relation between parental mental state language and ToM. Besides, we considered different specific indicators of the home language and literacy environment as well as children’s language and ToM within one longitudinal study. In particular, we analyzed how quantitative and qualitative aspects of language and literacy stimulation at home relate to the children’s language development, and also whether and how these effects transfer to a domain that is closely related to language skills in development, namely ToM understanding. As parental mental state language can be conceptualized as a specific facet of the home language and literacy environment, we also analyzed whether and how this specific facet is related to more general facets of the home language and literacy environment and the development of language skills.

In line with numerous other studies, our study supports the assumption that the language and literacy stimulation at home is significantly related to children’s language development. Further, our results provide evidence that general aspects of the home language and literacy environment are also related to the developing ToM understanding in childhood. Consistent with our results, Boerma et al. (2017) found book exposure at home to be related to both language skills and ToM understanding in children aged 9–10 years. Similarly, Adrian et al. (2005) reported the quantity of joint picture book reading to be related to ToM in preschool children. Our study adds to these results by showing that the effect of book exposure at home unfolds its effects on ToM through children’s language development in preschool years.

Contrary to our results concerning book exposure as a measure of the quantity of language and literacy stimulation at home, we did not find a significant effect of the quality of verbal interaction on children’s language skills in the whole group but instead found a marginally significant effect on ToM understanding. However, this result does not imply that qualitative aspects of the verbally stimulating home learning environment are irrelevant to language development. Numerous studies, not least intervention studies, convincingly demonstrate that the quality of verbal interaction, e.g., during joint picture book reading, is highly relevant in promoting children’s language development (e.g., Whitehurst and Lonigan, 1998; Huttenlocher et al., 2010; Mol and Neuman, 2014).

One explanation why we did not find an effect of the quality of verbal interaction in our study may be that “the quality” of verbal interaction may not exist. Instead, specific aspects have been suggested and empirically demonstrated to promote different language skills or subdomains such as vocabulary, grammar, and early reading. Thus, drawing on data of the same comprehensive study as the present study, Lehrl et al. (2012) showed that the quality of verbal interaction significantly predicted the development of receptive vocabulary between ages 4 and 6, but not the children’s acquisition of receptive grammar; yet book exposure was related to changes in receptive grammar. Other studies (e.g., Huttenlocher et al., 2002) add to this by showing that the complexity of language input during mother-child interaction relates to changes in receptive grammar. This supports the assumption that specific aspects of the home language and literacy environment might be particularly relevant to specific subdomains of language development, such as grammar and vocabulary (see for a similar discussion also Ebert et al., 2013; Weinert and Ebert, 2013, 2017). As the various facets of the home language and literacy environment are only low to moderately correlated, averaging across them as well as across language domains may underestimate the specific impact of the quality of the home language and literacy environment. Indeed, when we differentiate between vocabulary and grammar, we find slightly different relations of the home language and literacy environment with the two language components (see Supplementary Figure B). For example, stronger correlations between book exposure and grammar than between book exposure and vocabulary show up. We also find a small effect of the quality of verbal interaction on the change of grammar between ages 4;6 and 5;6 but not on vocabulary.

Another explanation why our study did not reveal significant effects of the quality of verbal interaction on children’s language might be due to the fact that the quality of interaction was observed and rated within a rather short joint picture book situation. This situation might have been too short to capture the most relevant aspects of language stimulation. However, several studies have demonstrated that such time-economic measures, even some with less observational time (3 min), lead to valid results (e.g., Hindman et al., 2008; Landry et al., 2012). In addition, the substantial variance in our measure underpins that we were able to capture differences in interaction quality across families. Through implementing a multimethod-design and using observations and questionnaires, we also reduced possible methodological bias. However, as book exposure and mental state language input were measured via parent questionnaire, social desirability cannot be ruled out.

Altogether, our study contributes new evidence to previous studies by showing that the general characteristics of the home language and literacy environment are also relevant to other domains of children’s development, such as their ToM understanding. The effects of the home language and literacy environment on ToM thereby seem to unfold via children’s language development. Thus, our study adds to others that also show that the effects of the home language and literacy environment generalize to other domains of development via children’s language skills. For example, Daneri et al. (2018) documented that differences in the children’s vocabulary at 3 years of age mediated the relation between maternal language input and children’s executive functions at 4 years of age (with maternal language input being measured by the number of different words and the mean length of utterances during joint picture book reading). Another example is the study by Rose et al. (2017) which demonstrated that language skills partly mediate the relation between aspects of the home language and literacy environment and socioemotional development in children between the ages of 3 and 8 years. Thus, together with these results, our analyses again highlight the importance of the home language and literacy environment for children’s language development with far-reaching direct and indirect effects into other domains.

### SES and Children’s Language and ToM Development: The Mediating Role of the Home Language and Literacy Environment

In line with previous studies, our results indicate SES-related differences in children’s language (see Hoff, 2013) and ToM development (see Devine and Hughes, 2016). As many studies before, we also found that, on average, children from lower SES families lag behind their peers growing up in families with a higher SES in their language and ToM development. One explanation for these SES-related disparities might be confounded differences in the quantity and quality of the home language and literacy environment. In this vein, our findings support previous results in showing that the quantity and quality of language and literacy stimulation at home are related to the families’ SES (e.g., Hoff, 2003). Thus, children growing up in higher compared to lower SES families experience a higher quantity of book exposure and a higher quality of language stimulation at home. However, parental mental state language as a specific facet of the home language and literacy environment was not related to SES. This evidence points to the relative independence of rather general and more specific facets of the home language and literacy environment. Whether parents provide their children with a rich and varied language environment at home seems to be somewhat independent of whether and how parents speak about the mental world. However, notice that also book exposure as a proxy for the quantity of language and literacy stimulation at home and our measure of the quality of verbal interaction are only slightly related to each other. This result suggests that parents who provide their children with access to literacy and the opportunity to engage with books are not necessarily the same parents who use other language stimulating strategies and activities that are known to promote children’s language development. This pattern of a rather low association between facets of parental language stimulation is also found in other studies and for other age groups (e.g., Rodriguez et al., 2009; Lehrl et al., 2012; Attig and Weinert, 2019). However, despite these low associations, the different facets of the more general language environment are all related to SES in the present study as well as in other studies (e.g., Attig and Weinert, 2019; Linberg et al., 2020).

As we used a rather global measure of SES (HISEI), which includes occupation, prestige, income, and education, this might, amongst others, explain why in our study only book exposure mediates – at least partly – the relation between SES and children’s language skills as well as ToM understanding. The correlation between the families’ HISEI and book exposure might be due to other facets of the SES than the correlation between SES and quality of verbal interaction, and only these facets might be especially crucial for SES-related disparities in language skills. However, the result that book exposure but not the quality of verbal interaction mediated effects of family background on children’s language skills was somewhat surprising as other studies found especially the quality of verbal interaction to account for the relation between SES and child development (e.g., Huttenlocher et al., 2002; Hoff, 2003; Mol and Neuman, 2014; Daneri et al., 2018). For instance, Mol and Neuman (2014) showed that particularly parents’ contingent responsiveness to their 5-year-old children during a book-sharing task mediated the effects of SES on receptive and productive vocabulary. However, the authors also found book access to account for SES-related differences in productive vocabulary. These results again suggest that for different language components or subdomains, different facets of the home language and literacy environment might be particularly important. Thus, our measure of the quality of verbal interactions as well as our measure of language skills might have been too global to find relations between the assessed and aggregated quality aspects of verbal interactions and the children’s language skills (see also section “Specific Effects of the Various Facets of the Home Language and Literacy Environment on Language and ToM”). As already mentioned, other studies (e.g., Huttenlocher et al., 2002; Rowe, 2012; Mol and Neuman, 2014; Hirsh-Pasek et al., 2015) also hint to the assumption that specific facets of parents’ language input and language stimulating parenting behavior are related to specific components of language and also account for SES-related disparities in the respective language component or subdomain.

However, we found a significant relation between book exposure as a very global measure of the quantity of language and literacy stimulation at home and children’s language skills, which also partly mediated SES-related differences. This, of course, could be due to other variables underlying this relation. Parents who provide their children with many books and who often read together with their children and for their own pleasure might use more complex grammar and a richer and more varied vocabulary. In addition, book exposure might not be just an indicator of the quantity of language and literacy stimulation at home (see Mol and Neuman, 2014) but also a measure of a specific facet of the SES that is related to child development. Thus, book possession in a family might indicate financial resources of the family that are invested in education. And it might be this investment in education that explains why children from higher SES backgrounds show comparatively more advanced language and cognitive skills (see also Conger and Donnellan, 2007). In this vein, Evans et al. (2010) showed that children growing up in families with many books are experiencing, on average, 3 years more of schooling than children from families with less books independent of the family’s SES. This suggests that book access might be not just a proxy for how parents promote children’s language and literacy, but books at home may also indicate a higher commitment to knowledge acquisition and in scholarly culture (see also Mol and Neuman, 2014).

Concerning children’s ToM understanding our results reveal that SES-related differences in ToM are completely mediated by book exposure and the children’s early language skills. Other studies found that SES-related differences in ToM are not completely explained by parents’ mental state language and children’s language skills (Devine and Hughes, 2016; Ebert et al., 2017). Our study complements these findings by showing that the more general language environment and a broader measure of language skills accounted for differences in children’s ToM development that are related to SES. This result suggests that SES-related differences in ToM understanding might - to a large degree - be due to differences in children’s language skills and their general home language and literacy environment.

In comparison, concerning language skills, the home language and literacy environment did not explain all SES-related differences in language skills. Even if various facets of the home language and literacy environment and earlier language skills are accounted for, relations between SES and child language were still reasonably high and significant. This result suggests that SES affects children’s language skills at 4;6 and 5;6 years over and above the variables included in our study and that other variables not measured in the present study additionally account for SES-related differences in children’s language skills. As already mentioned, more specific facets of the verbal input may explain SES-related differences in language skills even better. Also other variables that are suggested, e.g., by family stress and family investment models, such as parents’ wellbeing, could be associated with both SES and child development and thus play a role in their interrelation (e.g., Conger and Donnellan, 2007; Baydar et al., 2014; Raffington, 2018).

Overall research results on the effects of SES on children’s development indicate that these are multifaceted. The home language and literacy environment is only one factor that explains why children from different SES-backgrounds differ in their language skills. This also implies that the home language and literacy environment might be one starting point for reducing SES-related disparities in language development and thereby, as our study shows, also in other areas of development, such as in children’s ToM or social understanding.

### Parental Mental State Language and Its Effects on ToM Understanding and Language Skills

A novel approach of our study, which combined lines of research on ToM and language development, was that we also investigated how a specific facet of the home language and literacy environment, the use of mental state language in everyday conversations, is related not only to ToM but also to language development. We assumed that parents’ use of mental state language reflects a specific facet of the home language and literacy environment. Therefore we investigated its impact on children’s ToM *and* language development.

Different from the results of the meta-analyses by Tompkins et al. (2018), we did not find parental elaborated mental state language to be more strongly related to children’s ToM than parents’ non-elaborated mental state language. We found only parents’ non-elaborated mental state language to relate to children’s ToM understanding, and only when referring to children from lower SES backgrounds. This result, however, resembles the results of Ebert et al. (2017), who also found that in children from lower SES background, non-elaborated mental state language affects children’s ToM development between 3 and 5 years. However, the children who were included in that study were also part of the sample of the present study. Thus, the evidence is not independent. Nevertheless, in extension of Ebert et al. (2017), we showed that this result holds within an extended sample and even when controlling for children’s language skills and, more importantly, other facets of the more general home language and literacy environment.

These new results lead to the cautious assumption that specific relations exist between parental mental state language and children’s ToM development that go beyond the effects of the general home language and literacy environment parents provide to their children. The results also suggest that whether parents talk about mental states or not is a unique characteristic of parents’ verbal interaction with their child. The unique role of parental mental state language also becomes apparent in the low correlations between the quantity and quality of the home language and literacy environment and parents’ mental state language. However, this low correlation may also be partly due to methodological reasons and the fact that mental state language was not assessed in an observational situation; instead, parents had to self-evaluate what they might say in fictitious situations described in a questionnaire. This task might have been hard for some parents. However, there is evidence that parents can evaluate the use of their mental state language quite well with the MMSII. Slaughter and Peterson (2012) reported significant correlations between mothers’ self-reported elaborated mental state language in the MMSII and their elaborated mental state talk while narrating stories to their children. Additional evidence that parents’ mental state talk is a unique characteristic of parents’ talk with their children or when narrating stories and that this characteristic affects ToM development over and above more general facets of the home language and literacy environment is provided by Adrian et al. (2005). Different from the present study, the authors used a picture book situation while documenting mother’s mental state talk and showed that mother’s use of mental state terms explained variance in children’s ToM over and above the number of words the mothers used and the frequency of picture book reading at home. In a similar vein Ruffman et al. (2002) showed that mothers’ mental state language during a picture book task was correlated with preschoolers’ ToM even when other types of mothers’ utterances were accounted for.

In fact, a relation between parents’ mental state language and ToM may be more easily detected when mental state language is assessed in an interactive context. This assumption is supported by studies investigating children’s use of mental state language: Whereas studies that examined the use of mental state language in preschool children in interactive contexts often report a significant relation between ToM skills and the use of mental state language (e.g., Dunn et al., 1991; Hughes and Dunn, 1998), studies testing children in non-interactive tasks often failed to detect a reliable association between children’s mental state language and their ToM (e.g., Charman and Shmueli-Goetz, 1998; Longobardi et al., 2016).

Extending earlier studies on the relation between parents’ mental state language and ToM, we also considered how parental mental state language is related to their children’s language skills. We could not find significant relations within the whole group; however, when focusing on children from comparatively low SES backgrounds, we found that parents’ preference to explain and elaborate on mental states was related to children’s language skills. This result suggests that a preference for elaborated mental state language might indicate how parents communicate in general, how they explain relations, how talkative they are, and how often they use decontextualized language. These characteristics may dominate elaborated mental state language and thus may also explain the low correlation between non-elaborated parental mental state language and children’s language skills.

However, from a methodological point of view a problem of the MMSII is that the preference for elaborated mental state language and non-elaborated mental state language are not independent of each other: If parents choose non-elaborated mental state language as their preference, elaborated mental state language will necessarily get a lower rank. However, analyses where we considered elaborated and non-elaborated mental state language separately did not change our results.

The reason why we, in contrast to others, found non-elaborated mental state talk to be more strongly related to ToM compared to elaborated mental state language is still not clear. This result may be due to specific sample characteristics (see also Ebert et al., 2017) or may have cultural reasons. To further explore this issue, more research, including and comparing the MMSII with parents’ language use in everyday situations and from different SES backgrounds, is needed.

Most important, however, our study provides evidence that parents’ mental state language at home is an important facet of children’s home language and literacy environment. Moreover, this facet is specifically related to children’s ToM development over and above children’s language skills and more general features of the home language and literacy environment, particularly for children from lower SES families. Thus, parents’ mental state language is more than just a proxy for the quality of the home language and literacy environment but an additional facet of the home language and literacy environment that is important to children’s development over and above other more general facets of the home language and literacy environment.

### Relations Between the Home Language and Literacy Environment and Children’s Language and ToM Development in Children From Lower SES Homes

An additional question of our study that is particularly relevant to early intervention was, whether the pattern of relations shown for the whole group of children also holds for children from lower SES homes. Other studies have suggested that the effects of the home language and literacy environment may even be more pronounced in children from more disadvantaged families. Our results are in line with this assumption.

In particular, in the lower SES group, we found effects of all measures of the home language and literacy environment included in our study: Book exposure as a quantitative indicator, quality of verbal interaction, and elaborated parental mental talk were related to child language at age 4;6, which in turn was highly predictive for the children’s language skills one year later. Besides, book exposure had an additional direct effect on later language skills at 5;6 years of age. Furthermore, language skills at 4;6 years were significantly related to children’s later ToM understanding, and thus an indirect effect of the home language and literacy environment on later ToM via children’s language skills was shown. Not least, non-elaborated parental mental state talk exerted a direct effect on ToM in our model (see also discussion above).

These results show similarities and some differences to the pattern of relations observed in the whole group of children. Thus, the quality of verbal interaction and elaborated parental mental state talk significantly affected early child language, particularly in the low SES group. Concerning ToM, early child language mediated the effects of all the three indicators of the home language and literacy environment that impacted on child language, and non-elaborated mental state language showed an additional direct effect on ToM.

Overall the relational pattern suggests a strong impact of differences in the home language and literacy environment as well as in early child language on children’s language and ToM understanding in children from lower SES families. This result is in line with previous studies showing effects of the home language environment particularly in children from low SES families (e.g., Bradley et al., 2001; Shahaeian et al., 2018).

This comparatively strong impact of the home environment within the lower SES group compared to the whole group and the higher SES group may be due to various reasons.

Amongst others, global and more specific language stimulation, as well as qualitative and quantitative aspects of the home language and literacy environment might be particularly crucial for children with less advanced skills (see also Weinert et al., 2012). In fact, it is known that children from lower SES families usually lag behind in their language skills (Hoff, 2013).

Another reason might be that it is especially important what parents with a comparatively low SES do and how they interact with their children to compensate for reduced educational, cultural, financial, or social resources that often go along with low SES. Thus, lower SES families might not have the financial resources, the education, the cultural capital, or might not live in a region where it is easy for children to obtain rich stimulation in the surrounding area (e.g., Conger and Donnellan, 2007). Especially in the countryside, where a considerable part of our sample came from, there may be fewer opportunities to attend cultural activities or to visit a library, a zoo, a museum, or a theater than in a larger town or city. Thus, compared to children growing up in lower SES families, children from higher SES families might have additional resources or a higher availability of sources that might compensate for disadvantages in the home language and literacy environment. Against this background, it might be critical what language and literacy stimulation parents of children from lower SES provide to their children and that they offer them a rich and varied home language and literacy environment no matter what the socioeconomic circumstances are.

From an intervention point of view, it is highly relevant that all aspects of the home language and literacy environment may help to foster child development. In particular, the role of early language is highlighted by the results of our study as it impacts future language development and ToM. As our results suggest, it seems very promising to support the home language and literacy environment as a starting point to reduce SES differences. Our study shows that especially children from lower SES backgrounds can profit from a stimulating home language and literacy environment.

## Data Availability Statement

The raw data supporting the conclusions of this article will be made available by the authors, without undue reservation, to any qualified researcher.

## Ethics Statement

The present sample was part of a more comprehensive German longitudinal study on child development and educational processes. The study was funded by the German Research Foundation, and compliance with ethical standards was approved by the review process. Appropriate consent to take part in this study was obtained from parents, and all information provided was voluntary.

## Author Contributions

SE developed the research ideas of this manuscript, wrote the first draft of the manuscript and was responsible for the analyses. SL and SW revised drafts of the manuscript, and contributed in discussing analyses. All authors were involved in data collection, development of constructs of the study, contributed to the article and approved the submitted version.

## Conflict of Interest

The authors declare that the research was conducted in the absence of any commercial or financial relationships that could be construed as a potential conflict of interest.
