# Supplementary figures and images for: Differential Effects of the Home Language and Literacy Environment on Child Language and Theory of Mind and Their Relation to Socioeconomic Background
Source: Front Psychol. 2020 Oct 29;11:555654. doi: 10.3389/fpsyg.2020.555654 (PMC7658343; doi:10.3389/fpsyg.2020.555654)

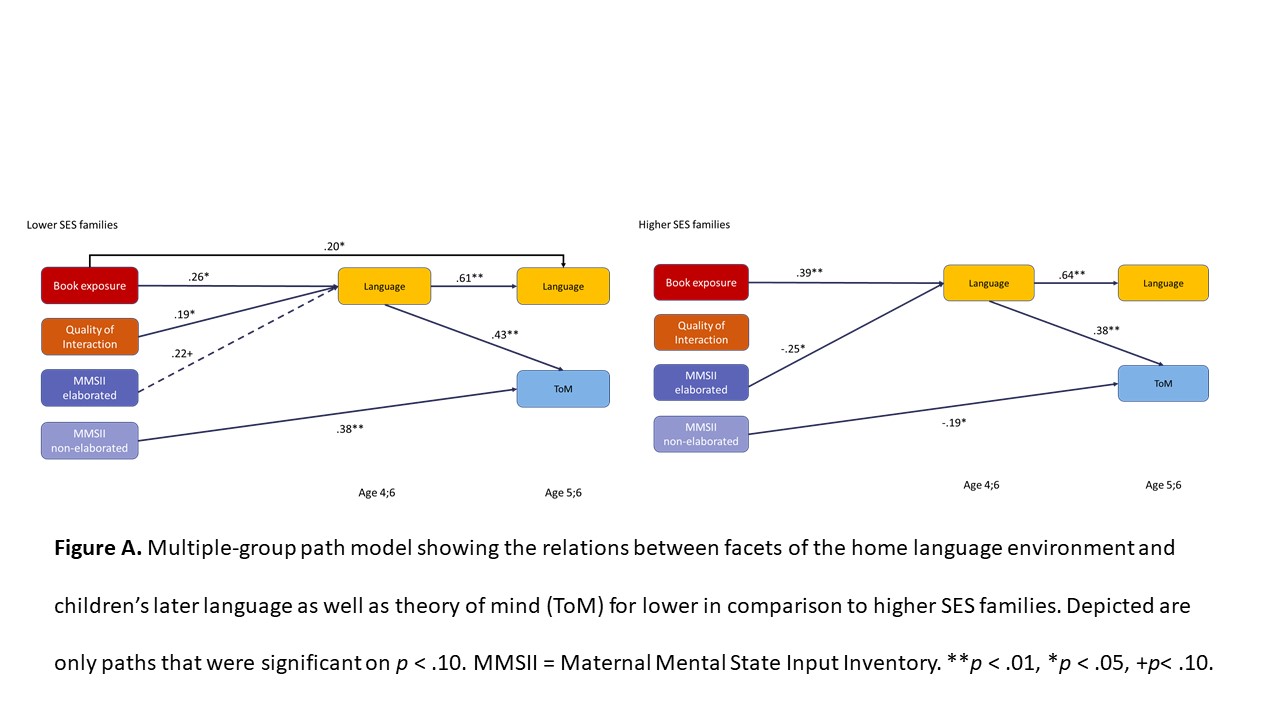

Supplement: Supplementary file 1 [file Image_1.jpg]

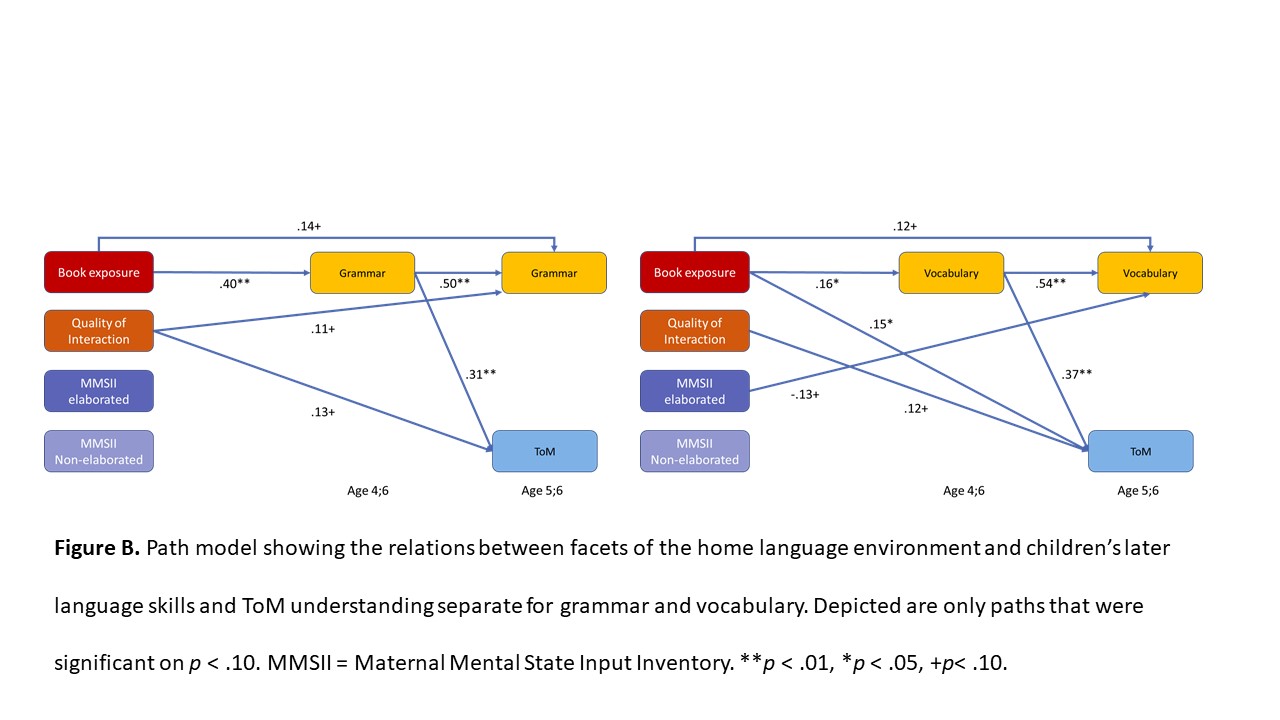

Supplement: Supplementary file 2 [file Image_2.jpg]
